# Antibiofilm Inhibitor Ferulic Acid as an Antibacterial Synergist Against *Escherichia coli*

**DOI:** 10.3390/biom15091253

**Published:** 2025-08-29

**Authors:** Zhijin Zhang, Jing Xu, Xiaojuan Wei, Rongbin Hu, Zhen Zhu, Zixuan Shang, Weiwei Wang, Bing Li, Yubin Bai, Jiyu Zhang

**Affiliations:** 1 Key Laboratory of New Animal Drug Project of Gansu Province, Key Laboratory of Veterinary Pharmaceutical Development of the Ministry of Agriculture, Lanzhou Institute of Husbandryand Pharmaceutical Sciences of CAAS, Lanzhou 730050, China; 2 College of Life Science and Food Engineering, Hebei University of Engineering, Handan 056038, China

**Keywords:** ferulic acid, *Escherichia coli*, antibiofilm, antibacterial synergist

## Abstract

*Escherichia coli* (*E. coli*) is a severe foodborne pathogen, and the formation of its biofilm can enhance bacterial virulence and reduce antibiotic sensitivity, posing a significant threat to human and animal health. Ferulic Acid (FA) is a natural active product that has been proven to possess various biological activities, including anti-inflammatory, antioxidant, and antitumor properties. This study evaluated the inhibitory effect of FA on the biofilm formation of *E. coli* through crystal violet (CV) staining and scanning electron microscopy (SEM) and investigated the synergistic effect of FA with antibiotics, using the alamar blue (AB) assay. In addition, the regulatory effect of FA on the transcription of biofilm-related genes was analyzed using qRT-PCR technology. The results showed that FA could significantly inhibit biofilm formation, reduce the production of extracellular polymeric substances (EPS), and weaken bacterial motility, without affecting bacterial growth and metabolic activity. qRT-PCR analysis revealed that FA significantly downregulated the expression of curli-related gene *csgD*, flagella-related genes (*flhC*, *flhD*, and *motA*), and type I fimbriae gene *fimA*, while upregulating the transcription of c-di-GMP-related genes (*pdeR*, *pdeA*, and *dosP*). It is noteworthy that FA exhibits significant synergistic antibacterial effects when combined with clinically commonly used antibiotics, including sodium fosfomycin, ceftriaxone, gentamicin, and tetracycline, with the most prominent synergistic effect observed in the combination of FA and sodium fosfomycin. These results confirm that FA possesses notable anti-biofilm activity and novel synergistic antibacterial properties, providing a potential therapeutic strategy for treating *E. coli* infections.

## 1. Introduction

Biofilms are highly organized microbial communities composed of bacterial cells and their secreted polymeric matrix [1]. This unique structure protects the internal microbiota through multiple mechanisms, forming a physical barrier that impedes the penetration of antimicrobial agents [2], while shielding against host immune system attacks and inducing microorganisms into a metabolically slow dormant state [3], thereby leading to severe treatment resistance and recurrent infections. Clinical studies have shown that infections caused by biofilm-producing bacteria often present more severe symptoms and significantly higher mortality rates [4]. In the medical field, biofilms related to medical devices are a major source of hospital-acquired infections [5], particularly those on urinary catheters and ventilator tubing. Due to the unique protective mechanisms of biofilms, such infections are often difficult to eradicate [6,7].

As a common commensal bacterium in the gastrointestinal tract, *Escherichia coli* (*E. coli*) is renowned for its robust environmental adaptability and diversity [8]. However, certain pathogenic strains of *E. coli*, particularly the O157:H7 serotype, can produce Shiga toxins, leading to severe symptoms [9]. These strains acquire multidrug resistance genes through horizontal gene transfer [10]. They are of particular concern due to their remarkable biofilm-forming capabilities; biofilm structures not only reduce the penetration rate of antimicrobial agents by more than 80%, but also effectively evade immune clearance, resulting in persistent infections [11,12,13]. According to the 2021 monitoring report by the European Food Safety Authority (EFSA), Shiga toxin-producing *E. coli* ranked third in the detection rate of pathogens among EU food enterprises, with the O157:H7 serotype posing the most severe public health risk [14]. This pathogen is primarily transmitted through the fecal–oral route, including contaminated food and drinking water, as well as cross-contamination during food processing through interpersonal contact or equipment surfaces [15]. Research indicates that *E. coli* biofilms may be present at various stages of food processing. These structured communities, composed of bacterial cells and their secreted exopolysaccharide (EPS), proteins, eDNA, etc., can increase bacterial resistance to disinfectants by up to 500 times, posing a continuous threat to food safety [16,17].

Given the limited efficacy of conventional therapies against biofilm-associated microbial infections, the development of novel alternative agents has become an urgent need. A key research focus in this field involves exploring natural bioactive compounds with anti-biofilm properties [18]. Studies have demonstrated that various plant-derived extracts and natural compounds can effectively inhibit bacterial biofilm formation through multi-target mechanisms, including interference with quorum-sensing systems and disruption of extracellular polymeric substances [19,20,21,22]. These compounds exhibit distinct advantages due to their multi-target action characteristics: they are less likely to induce bacterial resistance while showing potential as antibiotic adjuvants to enhance the therapeutic effects of conventional antimicrobial agents significantly. This approach offers promising solutions to address the declining efficacy of traditional antibiotics against foodborne pathogens such as O157:H7 serotype.

**Figure 1:**
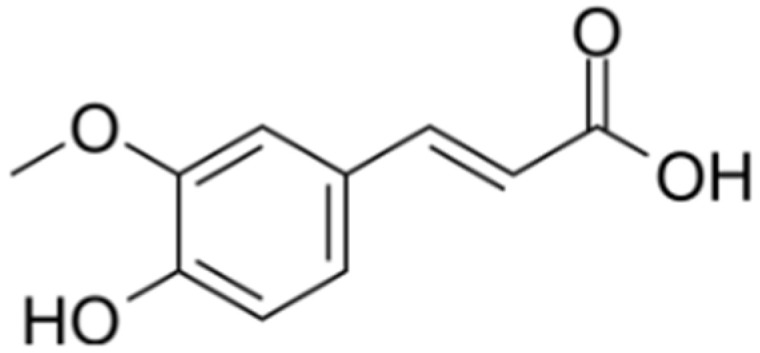
Ferulic acid chemical structure.

Ferulic acid (FA) is a phenolic compound widely found in fruits, vegetables, Chinese herbal medicines, and grains [23,24]. The phenolic hydroxyl and acrylic acid groups in its chemical structure convey significant biological activity (Figure 1). FA possesses a variety of biological activities. It not only acts as an antioxidant but also serves as an anti-inflammatory agent, capable of combating oxidative damage and mitigating inflammatory responses by scavenging excessive reactive oxygen species (ROS) or directly eliminating free radicals and free radical-generating enzymes [25]. Additionally, FA can exert antithrombotic effects by inhibiting platelet aggregation and the release of thromboxane-like substances [26], and it can reduce blood lipids by suppressing hepatic cholesterol synthesis [27], thereby preventing coronary heart disease and atherosclerosis [28,29]. Although there have been reports in the literature on the anti-biofilm activity of FA in recent years [30,31,32], research on its synergistic effects with antibiotics remains relatively limited. This study systematically elucidates the inhibitory effects of FA on *Escherichia coli* biofilms through in vitro experiments and evaluates its synergistic effects with conventional antibiotics.

## 2. Materials and Methods

### 2.1. Materials and Bacterial Strains

The FA used in this study was purchased from MedChemExpress, prepared as a 40 mg/mL stock solution using dimethyl sulfoxide (DMSO), and diluted to the required concentration during the experiment, with the final concentration of DMSO not exceeding 1%.

*E. coli* O157:H7 (ATCC 43895) was purchased from the American Type Culture Collection (ATCC). Luria–Bertani (LB, HuanKai Microbial, Guangzhou, China) and Luria–Bertani agar (LA, HuanKai Microbial, Guangzhou, China) media were used for the growth of *E. coli* strains.

Caco-2 cell lines were obtained from ATCC and cultured under standard conditions using MEM medium with 20% FBS, 1 mM sodium pyruvate, 1 mM L-glutamine, 10 mM HEPES, and 1% non-essential amino acids.

### 2.2. Growth and Metabolic Activity

The impact of FA on *E. coli* growth was investigated using the microbroth dilution method [33]. Bacterial suspensions containing different concentrations of FA were added to 96-well plates and incubated at 37 °C for 24 h, followed by optical density measurement at 600 nm using a microplate reader.

*E. coli* metabolic activity was analyzed by the Alamar Blue (AB) assay according to the previous method [34]. Briefly, bacterial cultures from each well were collected into sterile centrifuge tubes, washed three times with sterile PBS, and resuspended. Then, 90 µL of the resuspended bacterial solution was transferred to a 96-well plate, with 10 µL of AB reagent added to each well. After 1 h of incubation in the dark, absorbance was measured at 570 nm and 600 nm, using PBS containing only AB reagent as the blank control. Cellular metabolic activity was calculated using Formula (1). All tests were performed in triplicate.

(1) $$Metabolic\;activity\left(\%\right)=\frac{\left(E_{OXI\left(OD600\right)}\times T_{OD570}\right)-\left(E_{OXI\left(OD570\right)}\times T_{OD600}\right)}{\left(E_{red\left(OD570\right)}\times B_{OD600}\right)-\left(E_{red\left(OD600\right)}\times B_{OD570}\right)}\times 100$$

Eoxi(OD570) = 80,586: Molar extinction coefficient of oxidized AB at 570 nm;
Eoxi(OD600) = 117,216: Molar extinction coefficient of oxidized AB at 600 nm;
Ered(OD570) = 155,677: Molar extinction coefficient of reduced AB at 570 nm;
Ered(OD600) = 14,652: Molar extinction coefficient of reduced AB at 600 nm;
T: Test sample; B: Blank control.

### 2.3. Cytotoxicity

The cytotoxicity of FA on Caco-2 cells was assessed by measuring the activity of intracellular dehydrogenases using the CCK-8 method. Briefly, Caco-2 cells were seeded in 96-well plates at a density of 1 × 10^5^ cells/mL and cultured until the density of the cells reached approximately 80%. The cells were then treated with various concentrations of FA (3.125, 6.25, 12.5, 25, 50, 100, 200, and 400 µg/mL) for 24 h, with a blank control group without FA set up in parallel. After treatment, 10 μL of CCK-8 reagent (MCE, Shanghai, China) was added to each well, followed by incubation for an additional 1 h. The absorbance was measured at 450 nm, using a Multiskan Go microplate reader (Thermo Fisher Scientific, Waltham, MA, USA). All tests were performed in triplicate.

### 2.4. Biofilm Assay

#### 2.4.1. Biofilm Inhibition Assay

The biofilm biomass was quantified using the crystal violet (CV) staining assay [21,35]. Briefly, *E. coli* was statically grown for 24 h at 37 °C. Next, the cells were resuspended at OD600 of 1.0 and diluted about 100-fold. Subsequently, the diluted bacterial solution was mixed with FA in a white 96-well plate (Corning Costar^®^ 3599, Corning, Kennebunk, ME, USA). Cells underwent static incubation for 24 h at 37 °C, were washed three times with PBS (pH = 7.2) to eliminate the nonadherent cells, and fixed for 1 h at 60 °C. After fixation with methanol and staining with 0.1% CV for 30 min, the stained biofilm was rinsed with tap water to remove the dye that was not bound. The CV contained in biofilm was dissolved in 150 µL of 95% ethanol, and its absorbance at 595 nm was measured. All tests were performed in triplicate.

#### 2.4.2. Scanning Electron Microscopy (SEM) Analysis

The bacterial biofilm observation by SEM was performed according to previously described methods [36,37] with minor modifications. Briefly, bacterial suspensions containing different concentrations of FA were added to 96-well plates containing cell culture slides and incubated at 37 °C for 24 h. After incubation, the samples were washed three times with sterile PBS to remove planktonic bacteria and loosely adherent cells. The cell culture slides were then carefully removed from the wells and subjected to sequential fixation and gradient ethanol dehydration. Following gold sputter coating, the samples were examined using an FEI Versa 3D scanning electron microscope (Thermo Fisher Scientific).

### 2.5. EPS Production

The EPS production was quantitatively estimated by ruthenium red staining [38]. Briefly, overnight-cultured *E. coli* was diluted 1:100 in fresh LB broth and mixed with different concentrations of FA (400, 200, 100 μg/mL), and the bacterial solution without FA served as the positive control group. The mixtures were then added to a 96-well plate and incubated statically at 37 °C for 24 h. After washing three times with PBS, 200 μL of 0.01% ruthenium red solution was added to each well. A blank control contained only ruthenium red solution. The plate was incubated at 37 °C in the dark for 60 min. The residual staining solution was then transferred to a new 96-well plate, and the absorbance was measured at 450 nm using a microplate reader. The inhibition rate was calculated using Formula (2). All tests were performed in triplicate.

(2) $$EPS\;inhibitions\left(\%\right)=\frac{\left(AS-AP\right)}{\left(AB-AP\right)}\times 100$$

AB: Absorbance of the blank control solution, AS: Absorbance of the sample solution, AP: Absorbance of the positive control solution

### 2.6. Motility Assay

FA was evaluated for its effects on *E. coli* motility, as described earlier [21,39]. In brief, *E. coli* cultures overnight were adjusted to an OD600 of 0.01. A semisolid agar media (0.3% LB agar) containing 400, 200, and 100 μg/mL of FA was used for the motility assay. A 1 µL measure of the diluted bacterial solution was inserted into the middle of the plate and then incubated for 24 h at 37 °C. The size of the halo zone compared to the control was used to evaluate the motility. All tests were performed in triplicate.

### 2.7. qRT-PCR

The qRT-PCR [40] was performed to investigate the effect of FA on the transcription of biofilm-regulated genes in *E. coli*. *E. coli* was cultured with or without FA for 24 h at 37 °C. Total RNA was extracted using a Bacterial RNA Kit (Omega Bio-tek, Norcross, GA, USA). RNA concentration was measured using a NanoDrop OneC spectrophotometer (Thermo Scientific, Waltham, MA, USA), and RNA integrity was verified by agarose gel electrophoresis. Subsequently, cDNA was synthesized from RNA using the PrimeScript™ RT Reagent Kit with gDNA Eraser (Takara Bio, Kusatsu, Japan). qRT-PCR was carried out using TB Green^®^ Premix Ex Taq™ II (Tli RNaseH Plus) (Takara Bio, Kusatsu, Japan), and relative gene expression levels were calculated using the 2^−ΔΔCt^ method [41]. The 16s rRNA gene was used as an internal control, and the primer sequences used in this study are listed in Table S1.

### 2.8. Synergistic Antibacterial Activity of FA Combined with Antibiotics

The antibacterial efficacy of FA was evaluated using the previous method [21]. Briefly, overnight bacterial cultures were diluted to an OD600 of 0.01. The diluted bacterial suspensions were mixed with antibiotics (at 1/2 MIC, 1/4 MIC, and 1/8 MIC concentrations) in the presence or absence of FA (400 μg/mL), followed by incubation at 37 °C for 16–18 h. The metabolic activity of the mixed suspensions was then analyzed using the AB assay described in Section 2.2. All tests were performed in triplicate.

### 2.9. Statistical Analysis

The Student *t*-test and one-way ANOVA followed by Dunn’s multiple comparison test was performed to determine statistical significance, using GraphPad Prism 9.0. Data are shown as means ± SD. *p* < 0.05 was considered statistically significant.

## 3. Results

### 3.1. Effects of FA on Growth and Metabolic Activity of E. coli

**Figure 2:**
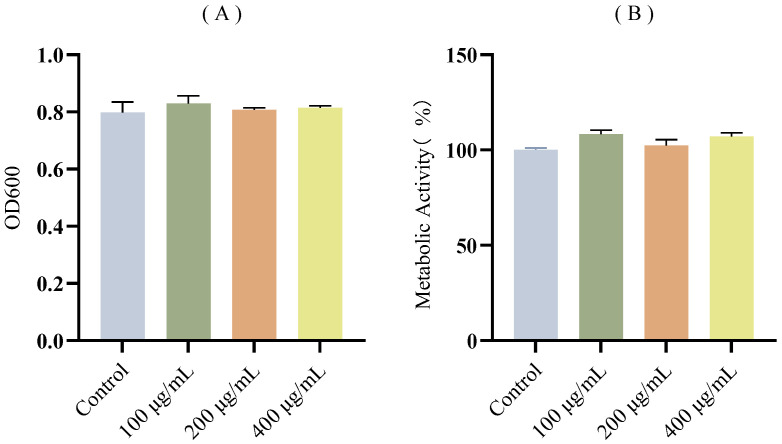
Growth and metabolic activity of *E. coli* in the presence of FA. (**A**) Effect of different concentrations of FA on *E. coli* growth. (**B**) Effect of different concentrations of FA on *E. coli* metabolic activity based on AB assay. All experimental results are presented as mean ± SD of triplicate determinations.

The effect of FA on growth and metabolic activity of *E. coli* was measured by microbroth dilution and AB assay. Compared to the control group, FA (100–400 μg/mL) showed no significant adverse effects on bacterial growth and metabolic activity (Figure 2). These results demonstrate the non-bactericidal nature of FA and its lack of alteration of metabolic activity in *E. coli*.

### 3.2. Cytotoxicity of FA on Caco-2 Cells

**Figure 3:**
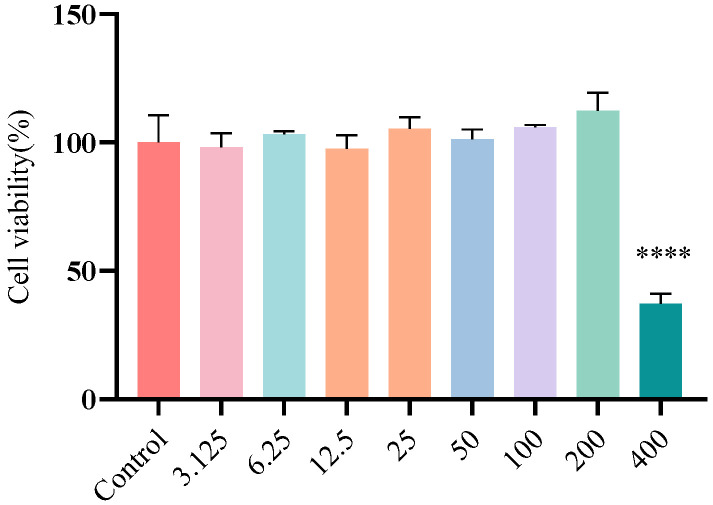
The cytotoxicity of FA in Caco-2 cells. FA was applied to cells at various concentrations (3.125, 6.25, 12.5, 25, 50, 100, 200, and 400 µg/mL) for 24 h. Results from all experiments are presented as the mean ± SD of three replicates. **** = *p* < 0.0001.

We systematically evaluated the cytotoxic effects of different concentrations of FA on Caco-2 cells using the CCK-8 assay (Figure 3). The results showed that when FA concentrations were at or below 200 µg/mL, there was no statistically significant difference in cell viability compared to the control group (*p* > 0.05), indicating no apparent cytotoxicity of FA within this concentration range. However, when the FA concentration increased to 400 µg/mL, cell viability significantly decreased by 62.77% (*p* < 0.0001), demonstrating cytotoxic effects.

### 3.3. The Effect of FA on E. coli Biofilm

**Figure 4:**
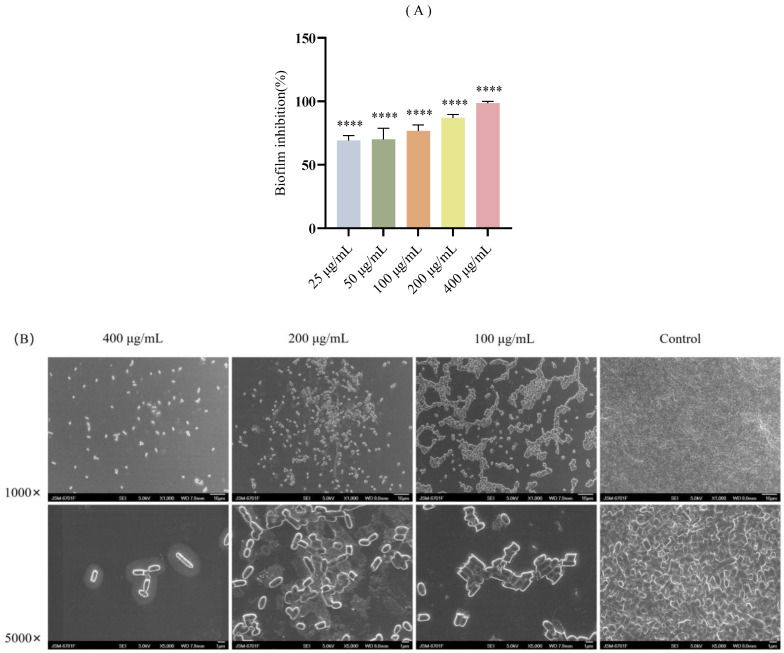
Effects of FA on *E. coli* biofilms. (**A**) Inhibition of biofilm formation by different concentrations of FA (25, 50, 100, 200, and 400 μg/mL) after 24 h treatment, **** = *p* < 0.0001, (**B**) SEM images of *E. coli* biofilms after 24 h treatment with different concentrations of FA (100, 200, and 400 μg/mL).

Different concentrations of FA were measured for antibiofilm activity by CV staining. This study showed that FA treatment significantly inhibited the formation of *E. coli* in a dose-dependent manner (Figure 4A, *p* < 0.0001). FA effectively prevented biofilm formation at concentrations as low as 25 μg/mL (inhibition rate = 69.28%). To further investigate the inhibitory effects of FA on biofilms, SEM was employed for imaging analysis. The results demonstrated that FA significantly inhibited biofilm formation (Figure 4B).

### 3.4. Effects of FA on EPS Production of E. coli

**Figure 5:**
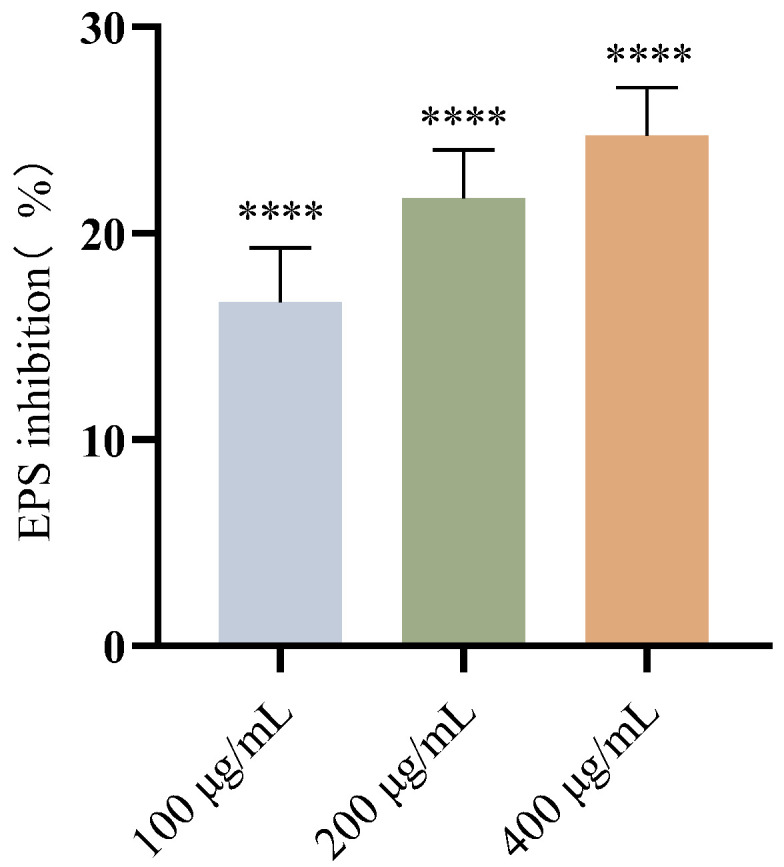
Results of EPS inhibition (%) at different concentrations of FA (100, 200, and 400 μg/mL) for 24 h. Results from all experiments are presented as the mean ± SD of three replicates. **** = *p* < 0.0001.

EPS serves as the structural scaffold of bacterial biofilms, encapsulating bacterial cells to form a three-dimensional architecture while providing protection to the bacteria [42]. EPS production in biofilms was studied using ruthenium red staining. The results demonstrated that FA inhibited EPS production in a dose-dependent manner (Figure 5, *p* < 0.0001). This finding aligns with the inhibitory effect of FA on *E. coli* biofilm formation.

### 3.5. Effects of FA on the Motility of E. coli

**Figure 6:**
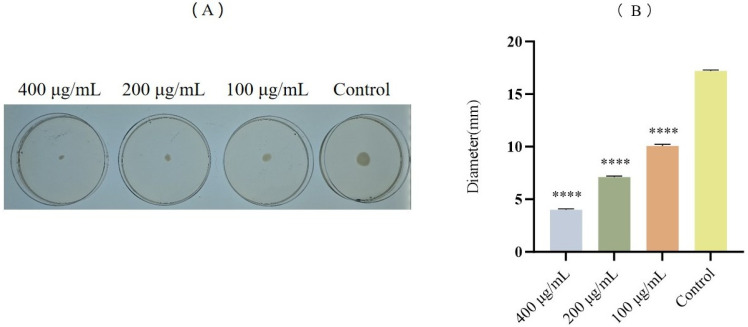
Inhibitory effect of FA on *E. coli* motility. (**A**) Motility images of *E. coli* following co-incubation with different concentrations of FA (100, 200, and 400 μg/mL), (**B**) Quantitative assessment of motility based on halo zone diameter. All experimental results are expressed as mean ± SD of triplicate determinations. **** = *p* < 0.0001.

Biofilm formation is closely associated with bacterial motility. Experimental results demonstrated that FA significantly inhibited *E. coli* motility in a dose-dependent manner compared to the control group (Figure 6A). Furthermore, quantitative analysis of the halo zone size revealed that all FA treatment groups exhibited significantly smaller inhibition zones than the control (Figure 6B, *p* < 0.0001).

### 3.6. Effect of FA on the Transcription of Biofilm-Regulated Genes of E. coli

**Figure 7:**
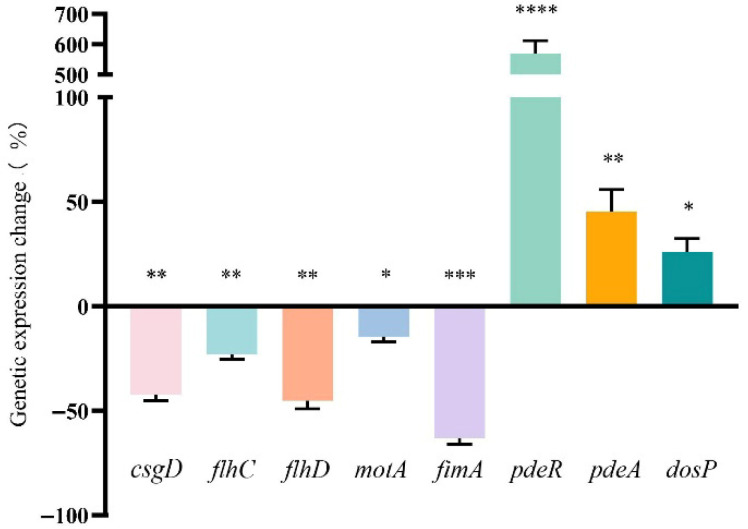
Effects of FA on transcription of biofilm-related genes. qRT-PCR analysis revealed significant transcriptional alterations in eight key genes (*csgD*, *flhC*, *flhD*, *motA*, *fimA*, *pdeR*, *pdeA*, and *dosP*) compared to the control group. * = *p* < 0.05, ** = *p* < 0.01, *** = *p* < 0.001, **** = *p* < 0.0001.

To investigate the molecular mechanisms underlying FA-mediated biofilm inhibition, we performed a qRT-PCR analysis of key biofilm-associated genes, including curli gene *csgD*, type I fimbriae gene *fimA*, flagellar-related genes (*flhC*, *flhD*, *motA*), and c-di-GMP-related genes (*pdeR*, *pdeA*, *dosP*). The results demonstrated that FA treatment significantly downregulated the expression of *csgD* (43.32% reduction), *flhC* (23.14%), *flhD* (45.36%), *motA* (14.62%), and *fimA* (63.24%). Conversely, FA markedly upregulated the transcription of *pdeR* (569.10% increase), *pdeA* (45.35%), and *dosP* (26.02%) (Figure 7).

### 3.7. Synergistic Effects of FA in Combination with Antibiotics Against E. coli

**Figure 8:**
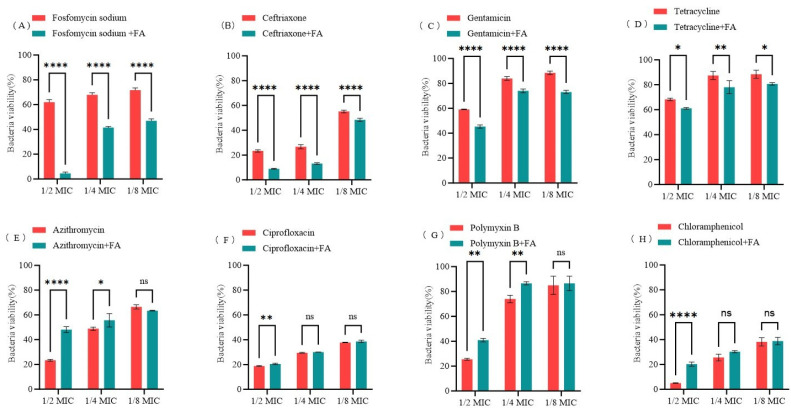
Effects of FA (400 μg/mL) combined with antibiotics on bacterial viability of *E. coli* O157:H7. (**A**) Fosfomycin sodium, (**B**) Ceftriaxone, (**C**) Gentamicin, (**D**) Tetracycline, (**E**) Azithromycin, (**F**) Ciprofloxacin, (**G**) Polymyxin B, (**H**) Chloramphenicol. All experimental results are presented as mean values ± SD from three independent replicates. ns = *p* > 0.05, * = *p* < 0.05, ** = *p* < 0.01, **** = *p* < 0.0001.

Using *E. coli* O157:H7 as the model strain, and based on changes in bacterial metabolic activity, we systematically evaluated the synergistic effects of 400 μg/mL FA with sub-inhibitory concentrations of antibiotics (1/2 MIC, 1/4 MIC, and 1/8 MIC) using the AB method. As illustrated in Figure 8, FA potentiated the antimicrobial efficacy of fosfomycin sodium, ceftriaxone, gentamicin, and tetracycline against *E. coli*. Notably, the most pronounced synergistic antibacterial activity was observed when FA was combined with fosfomycin sodium.

## 4. Discussion

The study demonstrates that FA significantly inhibits the formation of *E. coli* biofilms, the production of EPS, and motility, without affecting bacterial growth and metabolic activity. Notably, FA displayed synergistic antimicrobial effects when combined with various clinically relevant antibiotics, including fosfomycin sodium, ceftriaxone, gentamicin, and tetracycline. These findings suggest that FA not only possesses intrinsic antibiofilm properties, but also functions as an antibiotic adjuvant, thereby offering a promising combinatorial therapeutic strategy against *E. coli* infections.

As a crucial structural component of bacterial biofilms, EPS plays multifaceted roles in environmental adaptation and drug resistance development, by not only facilitating initial bacterial adhesion and colonization on abiotic surfaces but also enhancing bacterial survival through constructing three-dimensional matrix architectures, nutrient storage, and environmental stress resistance [43,44,45,46]. Our results demonstrate that FA exerts dose-dependent inhibitory effects on EPS production in *E. coli*, with corresponding dose-responsive suppression of biofilm formation and bacterial motility, suggesting that its antibiofilm mechanism may involve dual inhibition of EPS biosynthesis and motility. SEM further confirmed a substantial reduction in biofilm biomass on cell culture slides following FA treatment. These findings are consistent with the results reported by Pattnaik et al. [31]. Furthermore, Vaikkathillam et al. [47] reported similar antibiofilm activity against *Enterobacter hormaechei* and *Klebsiella pneumoniae*, potentially mediated through interference with adhesion molecules and modulation of c-di-GMP signaling. Collectively, these studies position FA as a promising natural compound for developing novel antibiofilm therapeutics.

To further investigate the mechanism underlying the anti-biofilm activity of FA, we analyzed the transcriptional levels of related genes using qRT-PCR. The results demonstrated that FA significantly downregulated the expression of curli-related gene *csgD*, flagella-related genes *flhC*, *flhD* and *motA*, and type I fimbriae-related gene *fimA*, while markedly upregulating the transcription of c-di-GMP metabolism-related genes (*pdeR*, *pdeA*, and *dosP*). The curli fibers regulated by *csgD* are crucial components of the extracellular matrix in biofilms of *E. coli* [40] and *Salmonella* [41]. When the expression of *csgD* is downregulated, the ability of bacteria to form biofilms is weakened. The fimbrial structural protein encoded by *fimA*, although varying among different bacteria, is closely associated with the formation of bacterial biofilms [48,49]. Studies have shown that the fimbriae encoded by this gene in different pathogenic bacteria exhibit similar biological functions: in *E. coli*, the type I fimbriae encoded by *fimA* mediate bacterial adhesion [48]; in *Porphyromonas gingivalis*, the long fimbriae it encodes are key mediators in the initial formation of biofilms [50], while in *Actinomyces oris*, the shaft fimbriae encoded by *fimA* are important components of type II fimbriae involved in biofilm formation [51]. It is noteworthy that when the expression of the *fimA* gene is inhibited, the biofilm-forming ability of these bacteria is significantly reduced. The findings of this study demonstrate that FA can inhibit the formation of *E. coli* biofilms by significantly downregulating the expression of *csgD* and *fimA*. The motility of bacteria is closely related to their biofilm formation—a decrease in motility significantly inhibits biofilm formation [52,53]. As the key organ for bacterial movement, flagella play a decisive role in the initial contact between cells and surfaces and in the early stages of biofilm formation [54,55]. In *E. coli*, when the flagellar structure is defective or the motility function is impaired, the early stages of biofilm formation are severely hindered, further confirming that motility is a critical factor influencing the early stages of biofilm formation [56]. The downregulation of *flhC*/*flhD*/*motA* indicates that FA can effectively inhibit the expression of flagella-related genes, thereby reducing bacterial motility, a result that has been verified in semi-solid agar motility experiments. The formation of bacterial biofilms is typically regulated by the second messenger c-di-GMP, whose intracellular concentration controls the transition between the biofilm state and the planktonic state in bacteria [57]. Research has found that c-di-GMP is widely present in various bacteria, including *E. coli* [58], *Pseudomonas aeruginosa* [59], *Vibrio cholerae* [60], and *Vibrio parahaemolyticus* [61], among others. The concentration level of c-di-GMP exhibits a significant positive correlation with the extent of biofilm development: an increase in its concentration promotes the synthesis of adhesins and extracellular polysaccharides, thereby enhancing biofilm formation; conversely, a decrease in concentration enhances bacterial motility and leads to biofilm dissociation [62,63,64]. Zhang [65] et al. found that coumarin reduces the intracellular concentration of c-di-GMP by affecting the expression of c-di-GMP-related genes, thereby inhibiting the formation of *Pseudomonas aeruginosa* biofilms. This study found that FA significantly upregulates the expression of genes *pdeR*/*pdeA*/*dosP*, which encode phosphodiesterases (key enzymes that degrade c-di-GMP) [66,67], leading to a decrease in intracellular c-di-GMP levels and consequently affecting the formation of *E. coli* biofilms. In summary, FA likely exerts its anti-biofilm effects through a dual mechanism: (1) by suppressing the expression of fimbriae- and flagella-related genes, thereby weakening bacterial adhesion and motility; and (2) by promoting c-di-GMP degradation, reducing its intracellular concentration, and consequently inhibiting biofilm formation.

Biofilm formation represents a critical factor contributing to chronic infections and antibiotic treatment failure [68]. In recent years, as biofilm-associated infections caused by multidrug-resistant bacteria have become increasingly prevalent, strategies utilizing either synthetic or natural bioactive compounds to inhibit or disrupt biofilms while enhancing the efficacy of antibiotics have demonstrated unique advantages [69,70,71,72,73]. Studies have shown that ionic liquids exhibit synergistic antibacterial activity with various antibiotics. Yang [74] et al. discovered that ionic liquids have synergistic antibacterial activity with multiple antibiotics (tetracycline, doxycycline, chloramphenicol, kanamycin, etc.), and their combination with antibiotics can effectively reduce the concentration of antibiotics required to inhibit microbial growth. Florio [75] et al. evaluated the interactions between colistin and ionic liquids with Gram-negative bacteria through a checkerboard assay. The results revealed that the ionic liquids 1-methyl-3-dodecylimidazolium bromide, 1-dodecyl-1-methylpyrrolidinium bromide, and 1-dodecyl-1-methylpiperidinium bromide all exhibited synergistic effects with colistin, demonstrating potential against infections caused by multidrug-resistant Gram-negative bacteria. Additionally, numerous natural products have also been shown to exert synergistic effects with antibiotics. For example, Bai [21] et al. found that ginkgo biflavone, as a biofilm inhibitor of *E. coli*, significantly enhanced the antibacterial activity of gentamicin, colistin B, and colistin E against drug-resistant *E. coli*. Many other natural compounds, such as carvacrol [69], quercetin [76], and baicalein [77], have also been demonstrated to possess anti-biofilm and synergistic antibacterial activities. In the present study, in vitro assessments of FA combined with various antibiotics revealed its capacity to markedly suppress the metabolic activity of *E. coli* when co-administered with fosfomycin, ceftriaxone, gentamicin, and tetracycline. Notably, the FA-fosfomycin combination exhibited the most potent synergistic antibacterial activity. Such natural product-based combination strategies may provide potential solutions for addressing the growing challenge of biofilm-associated infections.

## 5. Conclusions

Our findings demonstrate that FA exhibits significant antibiofilm and synergistic antimicrobial activities against *E. coli*. Experimental evidence confirms that FA effectively inhibits biofilm formation without compromising bacterial growth and metabolic activity. Furthermore, this compound dose-dependently reduces bacterial motility and EPS production. It is noteworthy that FA exhibits synergistic antibacterial activity with fosfomycin sodium, ceftriaxone, gentamicin, and tetracycline, particularly with fosfomycin sodium. Based on these results, we propose that FA, as a naturally derived biofilm inhibitor, represents a promising candidate for combination therapy with existing antibiotics, potentially serving as a novel therapeutic strategy against biofilm-associated, drug-resistant *E. coli* infections.

## Data Availability

The data that support the findings of this study are openly available in figshare at http://doi.org/10.6084/m9.figshare.29825915.

## Supplementary Materials

The following supporting information can be downloaded at: https://www.mdpi.com/article/10.3390/biom15091253/s1, Table S1: qRT-PCR primer sequences.
