# Supplementary material for: Antibiofilm Inhibitor Ferulic Acid as an Antibacterial Synergist Against Escherichia coli
Source: Biomolecules. 2025 Aug 29;15(9):1253. doi: 10.3390/biom15091253 (PMC12467221; doi:10.3390/biom15091253)
Supplement: Supplementary file 1 [file biomolecules-15-01253-s001.zip › biomolecules-3830932-supplementary.pdf]

Table S1
qRT-PCR primer sequences

**Primer Names**	**Sequences（5' to 3'）**
csgD-F	AATCGCTGGCAATTACAGG
csgD-R	CCGCTTCCATCATATCCAG
flhC-F	ATGCTGCCATTCTCAACCGACTG
flhC-R	CGCATCGACGCCATTACACAAAC
flhD-F	CGTTAGCGGCACTGACTCTTCC
flhD-R	TTGCGTCAACTGAGTAATCGTCTGG
motA-F	GAAGCCTTGGAGCACTCTATCAACC
motA-R	CTTTGGTGTATTTGGAGCGACGAAAC
fimA-F	GCAGAGGTGTCATTATATCCC
fimA-R	CGTTCAGTTAGGACAGGTTC
pdeA-F	CCGATCTGGCGAAAGCGAAGTC
pdeA-R	TGCGGGCGACCAATCAAATACC
pdeR-F	CGGCTCCCCTTTCGCATTGG
pdeR-R	TGTCCTCGTCCGCCTTCCTTC
dosP-F	GCGGTAACGGCTGGCAGTTC
dosP-R	GCGAAGATTTGCGGCTGGTAAAC
16s RNA-left primer 1	GTGAAGTCATGCCAGGAGCT
16s RNA-right primer 1	CGAAGTATGCGTCCGGATCA
